# Etoposide, cisplatin, and sintilimab combined with anlotinib in successful treatment of adrenocortical carcinoma with lung metastasis: a case report

**DOI:** 10.3389/fonc.2024.1403762

**Published:** 2024-08-16

**Authors:** Wenjing Niu, Haimei Zhang, Xuezhen Ma, Hua Liang, Zhongshi Qiao, Zheng Wang, Lifeng Niu

**Affiliations:** ^1^ School of Clinical Medicine, Shandong Second Medical University, Weifang, China; ^2^ Department of Oncology, Qingdao Central Hospital, University of Health and Rehabilitation Sciences, Affiliated Qingdao Central Hospital of Qingdao University, Qingdao, China; ^3^ Department of Oncology, Zhucheng People’s Hospital, Weifang, China; ^4^ School of Clinical Medicine, Binzhou Medical University, Binzhou, China

**Keywords:** adrenocortical carcinoma, anlotinib, sintilimab, etoposide, cisplatin, case report

## Abstract

**Background:**

Adrenocortical carcinoma (ACC) is a rare malignant tumor that occurs in the adrenal cortex. It has a high degree of malignancy and comparatively poor overall prognosis. Surgery is the standard curative therapy for localized ACC patients. The combination regimen of etoposide, doxorubicin, cisplatin (EDP) plus mitotane has been considered as the standardized chemotherapy regimen for advanced ACC. However, new effective regimens are emerging for specific conditions in metastatic ACC.

**Case presentation:**

We report a case of a 66-year-old man diagnosed with metastatic ACC who had a large left adrenal mass (110 mm × 87 mm) and multiple metastases in both lungs. The patient was treated with EP and sintilimab for six cycles; anlotinib was introduced after the third cycle. Follow-ups after the second to fourth cycles found significantly reduced lung metastases with all imaging examinations indicating partial response (PR) status. The patient received maintenance therapy thereafter with sintilimab plus anlotinib. Until recently, the patient’s lung metastases and the left adrenal gland area mass (39mm × 29mm) have disappeared, and no disease progression has been observed. The progression-free survival of this patient has been extended to approximately 31 months, in sharp contrast to a median survival time of 12 months for majority of advanced ACC. The main adverse events during treatment were appetite loss and grade I myelosuppression and revealed only grade I hypertension and grade I hypothyroidism.

**Conclusion:**

This case highlights the remarkable response of our patient’s ACC to treatment with a novel combination of EP and sintilimab combined with anlotinib. Our findings suggest a safe and more effective combination therapeutic option for patients with adrenocortical carcinoma.

## 1 Introduction

Adrenocortical carcinoma (ACC) is a rare endocrine tumor with limited treatment options and a very poor prognosis (1). The annual incidence rate ranges from 0.7 to 2 per million people (2). Clinical manifestations of adrenocortical carcinoma are often atypical. Most ACC patients exhibit symptoms of hormone overproduction (such as virilism or Cushing’s syndrome) in approximately 60% of cases and pains in approximately 30%–40% of cases. Additionally, approximately 10%–15% of patients are incidentally diagnosed through imaging examinations (3). Clinical diagnosis includes imaging examinations and measurement of adrenal cortical hormone levels. Although surgery is the standard curative therapy for localized ACC patients (4), postoperative local recurrence and distant metastasis frequently occur. Radiation and ablative techniques have been utilized with variable benefit depending on the clinical scenario (4). Mitotane is the only adrenergic cytotoxic drug for the treatment of ACC by inhibiting mitochondrial respiration, causing mitochondrial membrane dysfunction and inducing endoplasmic reticulum stress, and it reduces the secretory function of ACC cells by inhibiting the function and expression of several enzymes in the adrenocortical steroidogenesis pathway (5). Mitotane is recommended as the first choice for adjuvant therapy by multiple international guidelines and consensus (2, 4). Adjuvant treatment with mitotane is recommended for completely resected tumors in stage III disease with potential residual lesions (R1 or Rx) or high-grade tumors (Ki67 >10%), while radiation therapy is generally considered ineffective for treating ACC (4). Generally, first-line treatment for advanced/metastatic disease is mitotane alone or mitotane in combination with chemotherapy. The combination regimen of etoposide, doxorubicin, cisplatin (EDP) plus mitotane has been considered as the standardized chemotherapy regimen for advanced ACC (6). Specifically, for those with advanced or recurrent ACC who cannot undergo the EDP-M protocol, findings from a limited phase II clinical trial suggest that a regimen of etoposide combined with cisplatin could be beneficial. Alternatively, a combination of etoposide + cisplatin + mitotane or cisplatin + mitotane alone may also be considered (7). Etoposide (VP-16) is a potent anti-tumor agent derived from lignans found in podophyllin. It specifically targets topoisomerase II during the S or G2 phase, leading to the formation of a stable cleavable complex (drug–enzyme–DNA), thereby inhibiting DNA repair and exerting its anti-tumor effects (8). Additionally, cisplatin, a platinum-based metal complex, acts as an alkylating agent primarily focused on DNA. It forms inter- and intra-strand crosslinks with DNA to create a DDP-DNA complex, interfering with DNA replication, affecting its synthesis, and promoting apoptosis in cancer cells (9). In summary, these two chemotherapeutic drugs target the G0/G1 phase of the cell cycle, interfere with DNA synthesis, and effectively kill or inhibit tumor cells. Other treatment strategies remain controversial.

Immune checkpoint inhibitors (ICIs) work by inhibiting the interaction between immune checkpoints, such as PD-1 and CTLA-4, and their ligands, thus preventing the immune escape of tumor cells, boosting the activity of T cells, and improving anti-tumor immune memory (10, 11). In recent years, checkpoint inhibitors targeting the PD1/PD-L1 or cytotoxic T-lymphocyte-associated protein 4 (CTLA-4) pathways have shown great success and have driven the development of immunotherapy (10). Sintilimab, a fully human high-affinity monoclonal antibody, is designed to target PD-1 and PD-L1. It works by inhibiting the interaction between PD-1 and PD-L1/PD-L2, thereby restoring the T-cell-mediated killing function of tumor cells (12).

Receptor tyrosine kinases (RTKs) play an important role in a variety of cellular processes including growth, motility, differentiation, and metabolism (13). Protein tyrosine kinases (PTKs) and protein tyrosine phosphatases (PTPs) regulate the activity of RTKs, and while normal tissues usually have no or low RTK activity, malignant cells often exhibit increased RTK activity and levels of oncogenic RTKs (14–16). Multiple powerful and well-tolerated tyrosine kinase inhibitors (TKIs) with single or multiple targets have been developed. Lenvatinib is a novel multi-targeted inhibitor of vascular endothelial growth factor receptors (VEGFRs), fibroblast growth factor receptors (FGFR), and proto-oncogenes RET and KIT, RET, c-Kit (17). Anlotinib is an oral multi-targeted anti-angiogenic TKI that inhibits VEGFRs 1–3, FGFR 1–4, epidermal growth factor receptor, platelet-derived growth factor receptor, and c-Met, with similar target spectrum to lenvatinib (18). During clinical application, chemotherapy is effective in directly eliminating proliferative malignant tumor cells while also presenting and activating tumor antigens, creating favorable conditions for immunotherapy (19). Additionally, the anti-vascular multi-target drug obstructs the immune supply of tumor cells and enhances the immune microenvironment, in combination with sintilimab to further activate T cells for an immune killing effect (19). The synergy of these three approaches can enhance their anti-tumor effects and complement each other, maximizing the overall anti-cancer impact. The panel failed to reach a conclusive agreement regarding the adjuvant use of cytotoxic drugs, considering their cardiotoxic side effects. Consequently, we decided to use the EP therapy. Additionally, since mitotane is not yet available in the mainland market of China, and the utilization of mitotane in the clinical treatment of ACC within the nation is limited, the EDP-M regimen was not chosen as the first-line treatment approach. Here, we report a case of a patient with malignant adrenocortical tumor and multiple lung metastasis successfully treated with EP and sintilimab combined with anlotinib.

## 2 Case report

**Figure 1:**
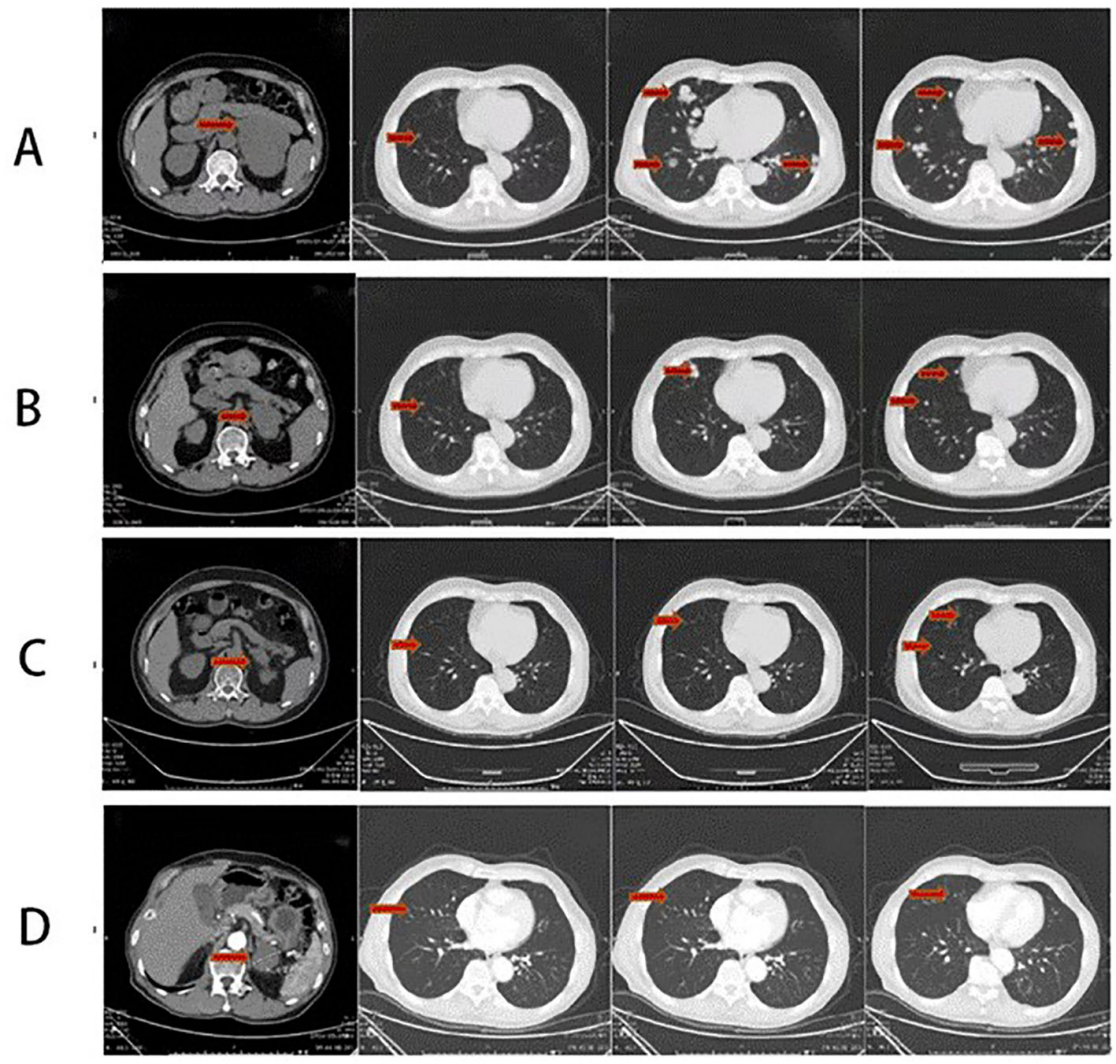
**(A)** Thoracic and abdominal CT scan before the therapy (2020-08) showing the following: mass in the left adrenal gland measuring approximately 110 mm × 87 mm suggestive of malignant tumor and multiple nodules in bilateral lungs suggestive of metastasis. **(B)** Thoracic and abdominal CT scan after four-cycle EP chemotherapy and anlotinib combined with sintilimab immunotherapy (2021-3): the left adrenal mass has significantly subsided, with a size of approximately 51 mm × 67 mm. Reduction in bilateral multiple pulmonary metastatic nodules. **(C)** Thoracic and abdominal CT scan after maintenance therapy for 8 months (2021-11): a mass in the left adrenal gland area with a cross-section of approximately 41 mm × 28 mm. The size of multiple bilateral lung metastasis further decreased. **(D)** Thoracic and abdominal CT scan after maintenance therapy for 24 months (2023-03): a mass in the left adrenal gland area with a cross-section of approximately 39 mm × 29 mm. The size of multiple bilateral lung metastasis further decreased. The whole red arrowheads show the before and after treatment of primary lesion of the left adrenal gland and the metastatic lesions of the lungs.

**Figure 2:**
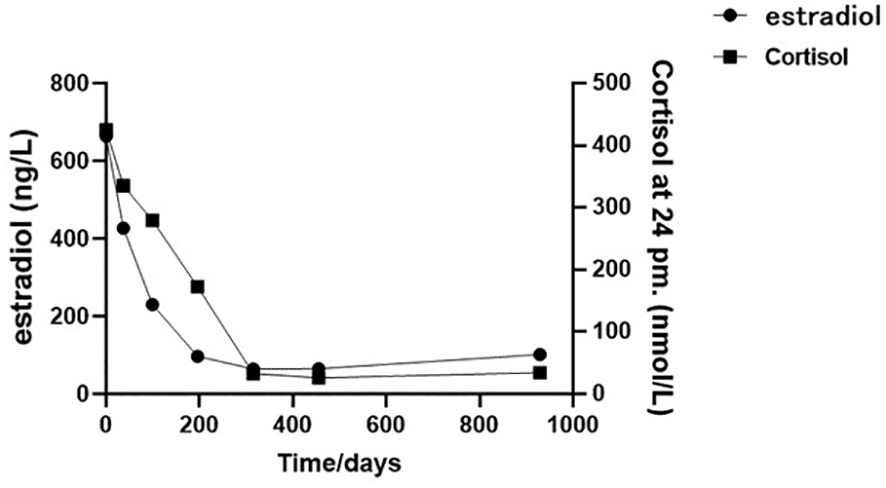
The cortisol and estradiol plotted against time (02/08/2020 to 04/03/2023): the initial sex hormone laboratory assessment was dated 02/08/2020, followed by subsequent assessments at approximately 1-month (23/09/2020), 3-month (24/11/2020), 6-month (01/03/2021), 9-month (13/06/2021), and 15-month (15/11/2021) intervals from the initial test, culminating in the final follow-up assessment in 04/03/2023.

The patient was a 66-year-old man from Shandong province, China. In August 2020, he complained of soreness in the left lower back, chest and gynecomastia, which worsens after physical activity, with no hypertension and electrolyte disturbance and without any history of glucocorticoid exposure. The patient was seen at our institution. Imaging examinations were implemented. Thoracic, upper and lower abdomen, and pelvic contrast-enhanced CT scan results showed multiple nodules in bilateral lungs suggestive of metastasis, enlarged mediastinal and right supraclavicular lymph nodes, and mass in the left adrenal gland measuring approximately 110 mm × 87 mm suggestive of malignant tumor (**Figure 1A**). The patient’s blood, urine, stool tests, and renal and liver functions were normal. The patient was placed in a supine position. Sex hormones examination showed testosterone of 1.03 ng/mL (reference range, 2.80–8.00 ng/mL), follicle-stimulating hormone <0.100 m IU/mL (reference range, <12.4 mIU/ml), luteinizing hormone of 0.25 mIU/mL (reference range, <8.6 mIU/mL), prolactin of 400.5 μIU/mL (referencerange, 86.00–324.00 μIU/ml), estradiol of 414.7 ng/L (reference range, 27.1–52.2 ng/L), and progesterone of 0.05 μg/L (reference range, 0–0.149 ng/L). Cortisol level was 538.7 nmol/L at 8 a.m. and 680.50 nmol/L at 12 a.m. (reference range, 118.60–610.00 nmol/L at 8 a.m. and 55–138 nmol/L at 12 a.m.), indicating excessive cortisol and disturbed rhythm. The patient’s adrenaline was 82.9 pmol/L (reference range, 0–605.9 pmol/L), noradrenaline was 2,243.2 pmol/L (reference range, 413.9–4,434.2 pmol/L), dopamine was 105.6 pmol/L (reference range, 0–196.00 pmol/L) (**Figure 2**). Due to financial reasons, the patient refused to undergo positron emission tomography/computed tomography (PET-CT) examination. After comprehensive consideration, we highly suspect that the patient has adrenal tumors.

**Figure 3:**
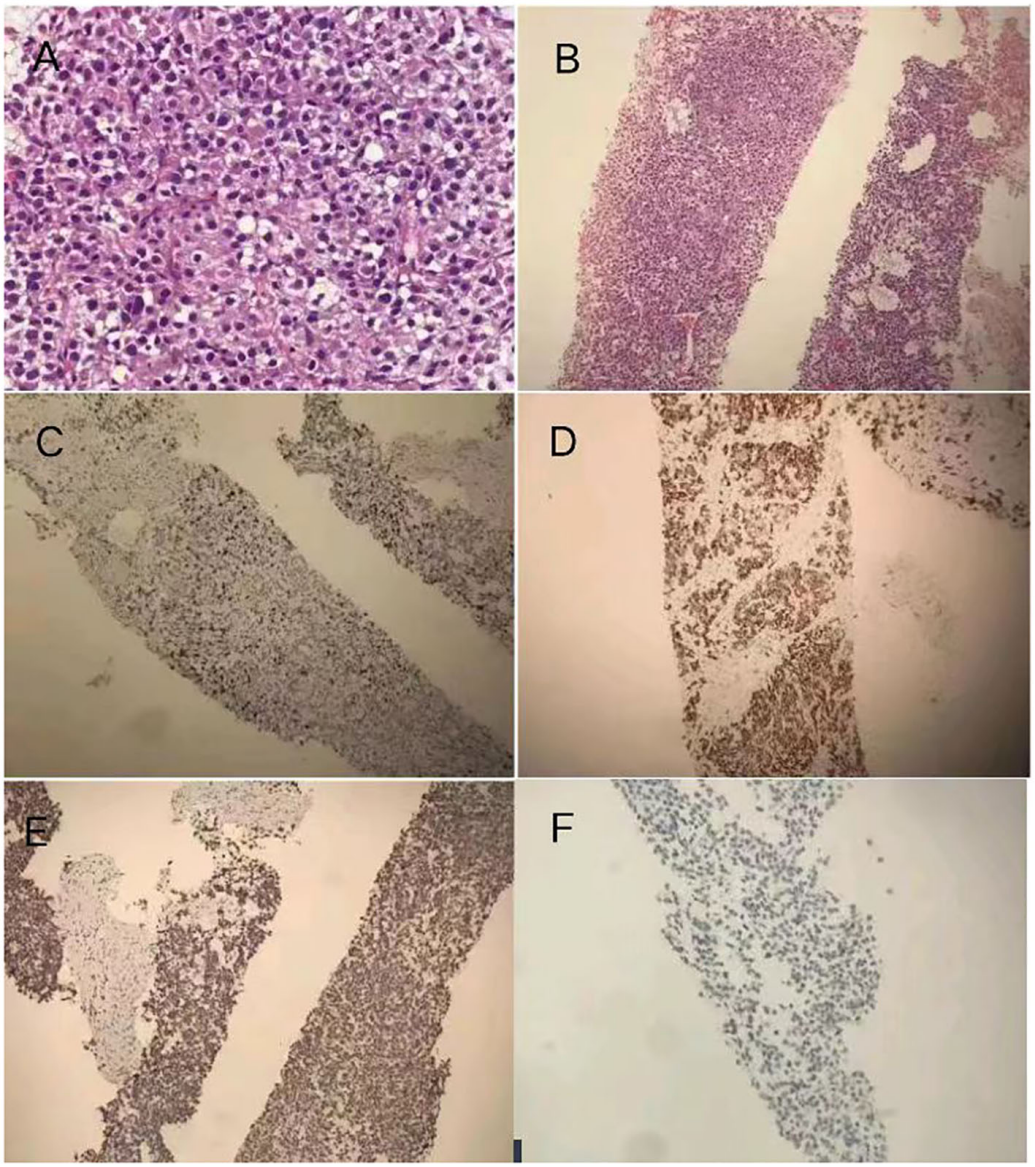
**(A)** Pathology and immunohistochemistry: the biopsy was performed on the mass in the left kidney area, which revealed pathological findings indicating malignancy likely originating from adrenocortical tissue. Immunohistochemistry results showing increased eosinophils with mucinous degeneration along with mild nuclear atypia and visible mitotic figures among tumor cells. **(B)** hematoxylin–eosin staining dyes ×100. **(C)** Positive expression of Melan-A in ACC section ×100. **(D)** Positive expression of Syn in ACC section ×100. **(E)** Positive expression of Ki-67 (approximately 10%+) in ACC section ×100. **(F)** Negative expression of RCC in ACC section ×100.

Under ultrasound guidance, a biopsy was performed on the mass in the left kidney area, pathologically revealing increased eosinophils with mucinous degeneration along with mild nuclear atypia and visible mitotic figures among tumor cells, which are rich in blood sinuses (**Figures 3A, B**). Immunohistochemistry results (**Figures 3C–F**) showed Inhibin (+), Melan-A (+), Syn (+), Vimentin (+), CK7 (−), CA-IX (−), CD10 (−), CK8/18 (−), TFE3 (−), CgA (partially weakly +), P504S (−), S-100 (−), Ki67 (approximately 10%+), and RCC (−). These results showed that the malignancy was likely to originate from the adrenocortical tissue. Additionally, the extended duration of tissue embedding and the formaldehyde crosslinking of samples significantly impacted the rate of DNA extraction, rendering the detection of mutations unfeasible; the patient’s peripheral plasma-free DNA (cfDNA) and peripheral blood gDNA were supplemented, revealing that no pathogenic or potentially pathogenic mutations were detected in the BRCA1/2 genes or other HRR pathway-related genes. The microsatellite stability (MSS) status was confirmed with a TMB of 0 mut/Mb.

**Figure 4:**
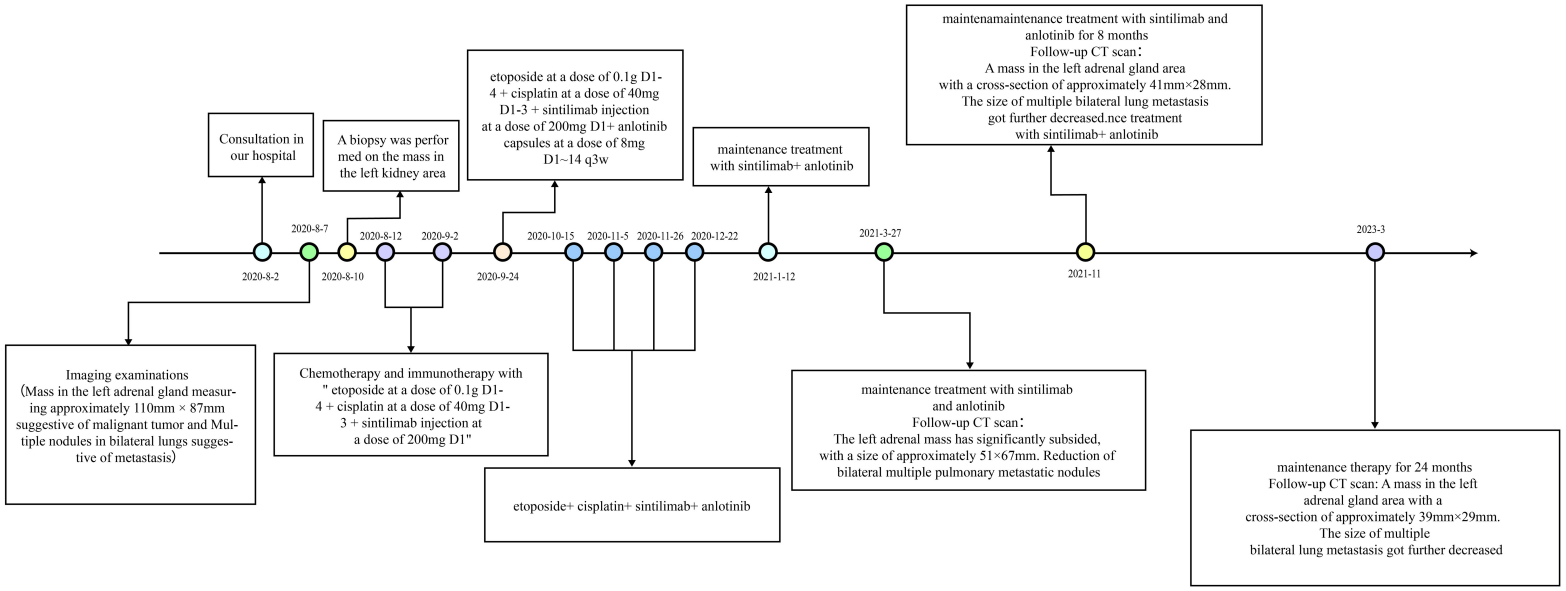
Treatment events timeline for the case: the patient was treated with EP and sintilimab for six cycles; anlotinib was introduced after the third cycle. Maintenance treatment with sintilimab and anlotinib lasted for 1 year.

The diagnosis was made accordingly as (left) adrenal malignancy with metastases in both lungs (cTxNxM1 stage IV). Considering the patient’s economic conditions and drug accessibility, we did not use mitotane for treatment. On 12 August and 2 September 2020, chemotherapy and immunotherapy with “etoposide at a dose of 0.1 g D1–4 + cisplatin at a dose of 40 mg D1–3 + sintilimab injection at a dose of 200 mg D1” were started. After two cycles of treatment, the patient still claimed a left flank pain and bilateral breast tenderness. From 24 September to 22 December 2020, the patient received “etoposide at a dose of 0.1g D1–4 + cisplatin at a dose of 40mg D1–3 + sintilimab injection at a dose of 200mg D1+ anlotinib capsules at a dose of 8mg D1~14 q3w” for four cycles (**Figure 4**).

In follow-up tests on 27 March 2021, the patient presented relief in bilateral breast tenderness. Follow-up CT scan (**Figure 1B**) showed significant reduction in size of adrenal gland mass and disappearance of some lung metastases, indicating partial response to treatment. The patient continued maintenance treatment with sintilimab and anlotinib. After a 10-month treatment, the follow-up outpatient CT scan on November 2021 compared to that on March 2021 showed a mass in the left adrenal gland area with a cross-section of approximately 41 mm × 28 mm. The size of multiple bilateral lung metastasis further decreased (**Figure 1C**). Sex hormones, including cortisol and estradiol, decreased over time to the normal range (**Figure 2**). The disease was re-evaluated as partial response (PR) according to RECIST 1.1 criteria. On 4 March 2023, the patient returned to receive treatment, and the therapeutic evaluation (**Figure 1D**) indicated stable disease (SD), without symptoms such as cough, chest tightness, or asthma.

The patient had been receiving regular maintenance treatment with sintilimab combined with anlotinib for 26 months, and the therapeutic evaluation indicated SD. By now, the progression-free survival (PFS) has reached 31 months, The primary side effects experienced during the treatment included decreased appetite following chemotherapy and grade I bone marrow suppression. Following the combined treatment of anlotinib and sintilimab, only grade I hypertension and grade I hypothyroidism were observed, but both were alleviated with symptomatic treatment using antihypertensive drugs and thyroxine tablets.

## 3 Discussion

To our knowledge, this study is the first clinical report on treatment of metastatic ACC with EP regimen combined with sintilimab and anlotinib. Complete surgical resection of the lesion is the only curative treatment for adrenocortical carcinoma (5). The combination of chemotherapy with mitotane was selected as the initial regimen following the ESMO Guidelines (5). To avoid the cardiotoxic effect of doxorubicin, we modified the EDP chemotherapy regimen to EP, which was shown to have comparable efficacy with advanced ACC (7, 20).

Immune checkpoint inhibitors such as anti-CTLA-4 antibodies and anti-programmed cell death protein 1 (anti-PD-1) antibodies and anti-PD-ligand 1 (PD-L1) have demonstrated antitumor activity in various solid malignancies, which has generated interest in their use for adrenocortical carcinoma patients (21). However, a phase II study on terminal ACC involving 39 patients found that MSI-H/MMR-D tumors are more common in ACC than has been recognized (22). Pembrolizumab is a novel monoclonal antibody that targets the interaction between programmed cell death protein 1 (PD-1) and its ligand (PD-L1) (23). Single-agent pembrolizumab has modest efficacy as a salvage therapy in ACC regardless of the tumor’s hormonal function, microsatellite instability status, or programmed cell death ligand-1 status (24–26) (**Table 1**). Sintilimab is a fully humanized immunoglobulin G4 anti-PD-1 monoclonal antibody developed in China (32), which targets PD-1 and PD-L1. Case reports have demonstrated the clinical efficacy of the EDP-M/EP regimen combined with Sintilumab in the treatment of advanced ACC, with good patient tolerance (33, 34). This suggests that a combination of EDP/EP-M plus sintilimab regimens may be a promising choice for the treatment of microsatellite stability status refractory ACC.

**Table 1:** Case series investigating immunotherapy or TKIs in patients with advanced adrenocortical carcinoma.

Characteristics	Cabozantinib	Pembrolizumab	Pembrolizumab	Pembrolizumab	Pembrolizumab	Nivolumb plus ipilimumb	Lenvantinib plus pembrolizumab	Cabozantinib or levantinib or pembrolizumab	Anlotinib with tislelizumab
n	16	2	6	1	39	1	8	15	37
≥ 2 lines of previous treatment	At least 10 (62%)	2 (100%)	1 (16%)	0 (0%)	28 (72%)	0 (0%)	8 (100%)	14 (93%)	21 (56.8%)
mPFS (months)	4	NR	NR	NA	2.1	NA	5.5	mTKI 6.3 Pem 1.4	8.2
mOS (months)	14.5	NR	NR	NA	24.9	NA	NR	mTKI 17.2 Pem 5.3	NA
Disease control rate	50%	50%	100%	0%	52%	100%	25%	mTKI 63% Pem 17%	78.4%
ORR	18.7%	50%	33%	0%	23%	100%	37.5%	mTKI 25% Pem 8%	35.1%
Outcome	PD 8 (50%) SD 5 (31%) PR 3 (18.7%) CR 0 (0%)	PD 1 (50%) SD 0 (0%) PR 0 (0%) CR 1 (1%)	PD 0 (50%) SD 4 (67%) PR 2 (33%) CR 0 (0%)	PD 1 (100%)	PD 15 (38%) SD 7 (18%) PR 9 (23%) CR 0 (0%)	PR 1 (100%)	PD 5 (62.5%) SD 1 (12.5%) PR 2 (25%) CR 0 (0%)	mTKI PD 3 (37.5%) SD 3 (37.5%) PR 2 (25%) CR 0 (0%) Pem PD 10 (83.4%) SD 1 (8.3%) PR 1 (8.3%) CR 0 (0%)	PD 8 (21.6%) SD 16 (43.2%) PR 12 (32.4%) CR 1 (2.7%)
Reference	Kroiss et al. (27)	Mota et al. (24)	Head et al. (25)	Casey et al. (26)	Raj et al. (22)	Nevgi et al. (28)	Bedrose et al. (29)	Miller et al. (30)	LI et al. (31)

With the development of molecular biology, molecular targeted therapy and medication have provided a more efficient and less toxic treatment approach for ACC. Embryonic adrenal angiogenesis is regulated by VEGF and Ang-Tie signaling pathways. VEGF angiogenic pathway was initially considered a promising therapeutic target for improving ACC prognosis (35). However, currently, their use in treating adrenocortical carcinoma only has theoretical feasibility. So far, no specific tyrosine kinase inhibitor has been approved for advanced ACC treatment (4). Preclinical studies have shown that anlotinib significantly inhibits cell migration and the formation of capillary-like tubes induced by VEGF/PDGF-BB/FGF-2 in endothelial cells *in vitro* and *in vivo*. A research into possible mechanisms indicated that anlotinib inhibits the activation of VEGFR2, PDGFRβ, and FGFR1, and downstream ERK signaling. The anti-angiogenic activity of anlotinib is stronger than that of three other anti-angiogenesis drugs, including sunitinib, sorafenib, and nintedanib (18, 36). Some studies reported the use of mTKI in advanced ACC pointing for a modest efficacy (27, 37, 38). Miller et al. found that among eight recurrent/metastatic patients who received single-drug MKI treatment (seven with lenvatinib and one with cabozantinib), the PR rate was 25%, including one patient lasting 23.5 months. Another three patients (38%) had SD; median PFS with single-agent MKI was 6.4 months (30). Multi-targeted anti-angiogenic TKI has shown good efficacy and manageable and expected safety in the treatment of advanced adrenocortical carcinoma. Based on our observations, we have noted that the lack of significant symptom remission in patients treated with immunotherapy alone is associated with the MSS status of patients. Therefore, we suggest that combining immunotherapy with molecular targeted therapy may be an effective treatment approach for these patients. Anti-angiogenic drugs can alleviate immune suppression, while immunotherapy can induce normalization of the vascular system or exert anti-angiogenic effects, which indicates a possible synergistic anti-tumor effect between immunotherapy and anti-angiogenic therapy (39). Studies showed that the combined therapy of anlotinib and ICIs in a neuroblastoma work, by promoting tumor vascular normalization at least in part via CD4 T cells, reprogrammed immunosuppressive TME to immunostimulatory TME, prolonging the period of vascular normalization and effectively preventing systemic immunosuppression (40). This could facilitate the distribution of chemotherapy-targeted drugs or immune checkpoint inhibitors within tumor tissues (41, 42). Moreover, it may downregulate PD-L1 expression on vascular endothelial cells through the inactivation of AKT pathway, thereby improving the ratio of CD8 T cells/FoxP3 T cells inside the tumor and remolding the immune microenvironment (43). A one-arm single-center clinical trial combining anlotinib with tislelizumab (humanized immunoglobulin G4 anti-programmed cell PD-1 monoclonal antibody) for metastatic adrenocortical carcinoma demonstrated that the objective response rate (ORR) was 35.1% (1 CR and 12 PR), the disease control rate (DCR) was 78.4% (29/37), the median PFS was 8.2 months (95% CI, 1.4–24.7), and the median OS was 30.6 months (95% CI, 21.1–NR) (31). These results indicate that the combination therapy of anti-angiogenic agents and PD-1 checkpoint inhibitors demonstrates clinical benefits with manageable toxicity (29).

Several prospective clinical trials for advanced ACC are underway (**Table 2**), there are no clinical studies reporting the combination of EP and sintilimab combined with anlotinib in the treatment of ACC. Therefore, the patient in this study signed the informed consent for off-label use of medication. Follow-up CT examination indicated complete disappearance of multiple lung metastasis and significant shrinkage of the primary adrenal lesion. So far, the patient has achieved PFS of almost 31 months and are now under surveillance. The expressions of TMB and MSI/MMR in this patient detection results may not fully reflect the molecular changes in the tumor tissue. Therefore, while this case report provides valuable insights, further exploration is needed to understand its specific mechanism. It is essential to validate these findings through studies with a larger sample size such as clinical trials to evaluate the efficacy of this combination therapy in a larger ACC patient population.

**Table 2:** Ongoing prospective clinical trials for advanced ACC as currently recruiting on ClinicalTrials.gov.

Drug/intervention	Study title	Setting	End point	Phase
Pembrolizumab + Mitotane	A Phase II Study to Evaluate the Efficacy and Safety of Pembrolizumab in Combination With Mitotane in Patients With Advanced Adrenocortical Carcinoma	1st (or later) lines	ORR	II
Pembrolizumab + Lenvatinib	Phase II Trial of Pembrolizumab Plus Lenvatinib in Advanced Adrenal Cortical Carcinoma	2nd (or later) lines	ORR	II
Cabozantinib	Cabozantinib in Advanced Adrenocortical Carcinoma	2nd line	PFS at 4 months	II
Cabozantinib	Cabozantinib in Treating Patients With Locally Advanced or Metastatic Unresectable Adrenocortical Carcinoma	2nd line	PFS at 4 months	II
Camrelizumab + Apatinib	Phase II Study for Combination of Camrelizumab and Apatinib in the Second-line Treatment of Recurrent or Metastatic Adrenocortical Carcinoma	2nd (or later) lines	ORR	II
Atezolizumab + Cabozantinib	Cabozantinib in Combination With Atezolizumab for the Treatment of Patients With Locally Advanced, Metastatic, or Unresectable Adrenal Cortical Cancer	1st line	ORR	II

## 4 Conclusion

In summary, we report a case of adrenocortical carcinoma successfully treated with EP plus sintilimab and anlotinib, which showed significant response with few signs of residual tumor viability in metastatic sites. Considering the limited treatment options and poor survival associated with ACC, the present case may provide a new insight for decision making of treatment modalities for ACC.

## Ethics declarations

The studies involving humans were approved by Clinical Medical Research Ethics Committee of Qingdao Central Hospital. The ethics approval number is KY202313601. The studies were conducted in accordance with the local legislation and institutional requirements. The participants provided their written informed consent to participate in this study. Written informed consent was obtained from the individual(s) for the publication of any potentially identifiable images or data included in this article.

## Rights and permissions

Open Access. This article is licensed under a Creative Commons Attribution 4.0 International License, which permits use, sharing, adaptation, distribution, and reproduction in any medium or format, as long as you give appropriate credit to the original author(s) and the source, provide a link to the Creative Commons license, and indicate if changes were made. The images or other third party material in this article are included in the article’s Creative Commons license, unless indicated otherwise in a credit line to the material. If material is not included in the article’s Creative Commons license and your intended use is not permitted by statutory regulation or exceeds the permitted use, you will need to obtain permission directly from the copyright holder. To view a copy of this license, visit http://creativecommons.org/licenses/by/4.0/. The Creative Commons Public Domain Dedication waiver (http://creativecommons.org/publicdomain/zero/1.0/) applies to the data made available in this article, unless otherwise stated in a credit line to the data. Reprints and permissions.

## Data Availability

The original contributions presented in the study are included in the article. Further inquiries can be directed to the corresponding author.
